# Safety observation of antiarrhythmic drug use in a patient with sinus bradycardia following atrial fibrillation radiofrequency ablation combined with cardiac neural ablation: a case report

**DOI:** 10.11604/pamj.2024.48.63.43881

**Published:** 2024-06-19

**Authors:** Qijun Zhang, Feiqin Shi, Bingjie Song, Yingchun Bao, Yong Cao

**Affiliations:** 1 Cardiovascular Department, The Affiliated People's Hospital of Ningbo University, Ningbo, 315000, ZheJia, People's Republic of China,; 2 Ningbo Yinzhou N°2 Hospital Community, Ningbo, 315143, ZheJia, People's Republic of China

**Keywords:** Atrial fibrillation, radiofrequency catheter ablation, cardiac ganglion ablation, antiarrhythmic drugs, sinus bradycardia, case report

## Abstract

This study assessed the safety of Antiarrhythmic Drug (AAD) administration in a patient experiencing sinus bradycardia following radiofrequency ablation for Atrial Fibrillation (AF), followed by cardiac ganglion ablation. Post-AF radiofrequency ablation, the employment of AADs is a prevalent clinical practice; however, these drugs may exacerbate bradycardia, leading to increased patient discomfort and treatment complexity. The decision to employ AADs in patients with sinus bradycardia post-AF ablation poses a significant clinical challenge. This investigation aimed to ascertain the safety of AADs in such patients. The study encompassed a single case, wherein a patient with pre- and post-procedure sinus bradycardia was treated with AADs following AF radiofrequency ablation and cardiac ganglion ablation, with a subsequent safety assessment. The findings indicate that AADs can be safely administered to patients with sinus bradycardia after these procedures, offering valuable insights for clinical decision-making. This case report underscores the intricacies of post-AF ablation management in patients with sinus bradycardia and advocates for personalized therapeutic strategies. The results enhance the clinical knowledge regarding the safety of AADs in this patient subset and may guide future treatment protocols. Nonetheless, the study's conclusions are drawn from a single case, and further research with larger cohorts is essential to substantiate these findings and elucidate the long-term safety and efficacy of this therapeutic approach.

## Introduction

Research on cardiac ganglion ablation for bradycardia is still emerging and may not be as extensive as research on other cardiac conditions like arrhythmias or hypertension. Nevertheless, certain studies have investigated the potential of cardiac ganglion ablation in managing bradycardia, especially when conventional treatments prove ineffective or impractical. Bradycardia is defined as a heart rate slower than normal, often less than 60 beats per minute. Symptoms such as dizziness, fatigue, and fainting can result, and in severe cases, intervention may be necessary to restore a normal heart rate. The rationale for employing cardiac ganglion ablation for bradycardia lies in its capability to modulate the activity of the cardiac nervous system [1]. Disrupting specific neural pathways within the cardiac ganglia may allow for heart rate adjustment and restoration of a more optimal rhythm. However, research in this area remains relatively limited, and additional studies are required to assess the efficacy, safety, and long-term outcomes of cardiac ganglion ablation for bradycardia specifically [2]. Clinical trials and observational studies are likely to evaluate the feasibility and effectiveness of this approach in various patient populations. Although there is interest in investigating cardiac ganglion ablation as a potential treatment for bradycardia, further research is required before widespread adoption in clinical practice for this indication.

Atrial fibrillation (AF) radiofrequency catheter ablation followed by the administration of antiarrhythmic drugs represents a common therapeutic approach. These medications serve to maintain sinus rhythm post-ablation, reducing the risk of AF recurrence and thereby enhancing patients' quality of life. However, it is worth noting that antiarrhythmic drugs may also induce bradycardia, thereby exacerbating patient discomfort and complicating treatment. For patients experiencing post-ablation bradycardia, the decision regarding the use of antiarrhythmic drugs becomes a challenging clinical dilemma. On one hand, abstaining from these medications may heighten the likelihood of AF recurrence, while on the other hand, their administration may lead to bradycardia and the emergence of prolonged pauses and additional clinical symptoms [3]. Though some studies and clinical practices have explored the use of antiarrhythmic drugs in patients with post-ablation bradycardia, research and clinical trials specifically targeting this scenario remain limited. Therefore, in clinical practice, physicians often rely on their own experiences and clinical guidelines to guide treatment decisions. This necessitates a careful balance between the benefits and drawbacks of antiarrhythmic drugs, taking into account individual patient circumstances and clinical requirements.

## Patient and observation

**Patient information:** in May 2023, a male patient was admitted due to recurring chest tightness and shortness of breath persisting for 2 months, worsening over the past day. His primary symptoms manifested as chest tightness experienced at home two months earlier, characterized by a squeezing sensation lasting approximately 30 minutes without any identifiable cause. The episode was accompanied by dizziness, a sense of object rotation, and alleviation upon rest. He promptly sought medical attention at another hospital. The electrocardiogram revealed paroxysmal atrial fibrillation, and the patient was discharged upon symptom alleviation. One day prior, the patient experienced a recurrence of chest tightness and shortness of breath, consistent with previous episodes. The patient promptly sought medical attention at our hospital. Considering the likelihood of coronary atherosclerotic heart disease, the patient was admitted to our department's outpatient service for further diagnostic clarification.

**Clinical findings:** the physical examination revealed the following vital signs: body temperature 36.4°C, pulse 45 beats/minute, respiration 19 breaths/minute, blood pressure 117/64 mmHg; alert and oriented, no signs of illness, no distension of the jugular veins, clear breath sounds bilaterally in the lungs without any dry or moist rales, heart rate 45 beats/minute, regular rhythm, other vitals were stable.

**Timeline of the current episode:** the patient was referred and admitted to our unit on 13^th^ May 2023. Coronary angiography was conducted on 15^th^ May 2023. Catheter ablation and anhydrous ethanol infusion VOM were done on 24^th^ May 2023. Hospital discharge on 27^th^ May 2023. Outpatient follow-up was carried out in June 2023 and January 2024.

**Diagnostic assessment:** the physical examination revealed the following vital signs: auxiliary examination results on May 12, 2023, indicated no abnormalities in our hospital's BNP, B-type natriuretic peptide at 18pg/ml, D-dimer, and myocardial three items. The electrocardiogram revealed sinus bradycardia with arrhythmia, characterized by a heart rate of 59 bpm. Over 24 hours, the patient's total heart rate was 69990 beats, ranging from a minimum of 37 bpm (2023-05-19 04: 56: 17) to a maximum of 76 bpm (2023-05-18 14: 57: 33), with an average of 49 bpm, and no prolonged intervals were noted. Echocardiography revealed a left atrial diameter of 26mm, left ventricular diastolic diameter of 50mm, systolic diameter of 35mm, septal thickness of 9mm, posterior wall thickness of 8mm, and an ejection fraction of 64%. Enhance pertinent investigations, including electrocardiography, complete blood count, troponin, blood biochemistry, echocardiography, and chest CT scans. If treatment contraindications are absent, administer aspirin and clopidogrel for antiplatelet aggregation, and atorvastatin calcium tablets to regulate lipid levels and stabilize plaques, aiming for a target LDL-C<1.8mmol/L. Symptomatic therapy may include ezetimibe in combination with lipid-lowering agents, deanxin for anxiety relief, and tanshinone for improving blood circulation. Coronary angiography should be conducted within 48 hours to confirm the diagnosis, with coronary intervention or coronary artery bypass grafting surgery considered as needed.

**Diagnosis:** 1) Paroxysmal atrial fibrillation; 2) Coronary atherosclerotic heart disease; 3) Insufficient cerebral artery blood supply; 4) Anxiety status; 5) Sinus bradycardia.

**Figure 1:**
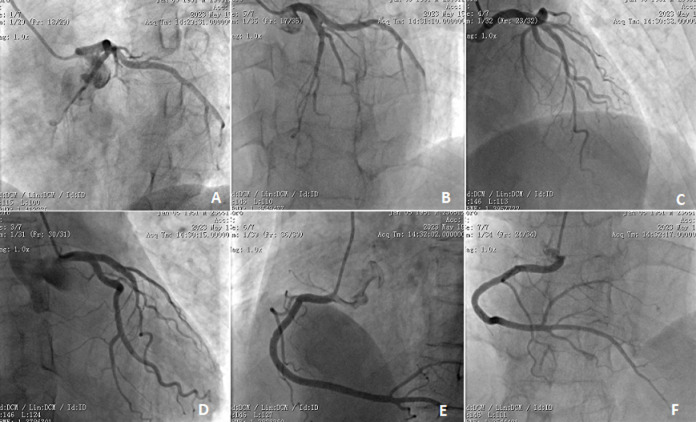
visualization of coronary angiography; A-D) left anterior descending artery (LAD) and the left circumflex artery (LCX) from different angles; E,F) right coronary artery (RCA) from varying perspectives

**Therapeutic interventions:** the result of coronary angiography conducted on May 15, 2023, revealed no stenosis in the left main trunk, 50% stenosis in the proximal segment of the anterior descending branch, myocardial bridging in the distal segment with 85% systolic compression, absence of significant stenosis in the circumflex branch, and 20-30% stenosis in the proximal and middle segments of the right coronary artery (Figure 1).

**Figure 2:**
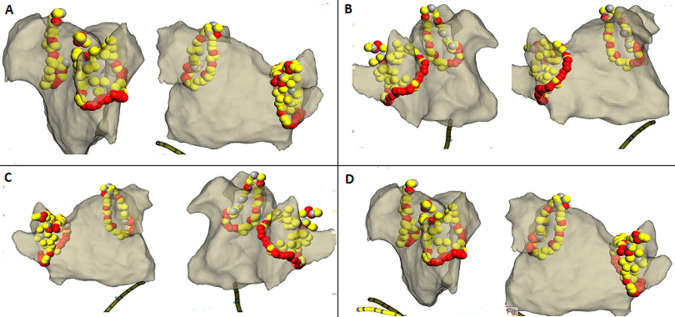
three-dimensional diagram illustrating successfully isolated pulmonary vein potentials: A,B) target map for ablation of the right pulmonary vein: (RL: P-A; RL: AP); (C,D) target map for left circumferential pulmonary vein ablation: (RL: P-A) (AP: LAO)

**Figure 3:**
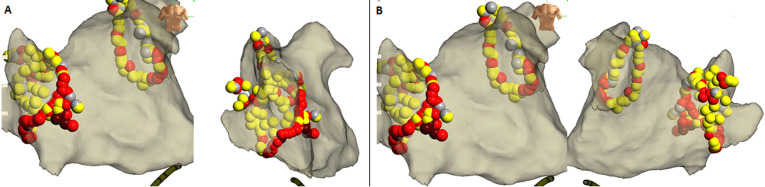
localization of the right atrial ganglionated plexus (RAGP); the red arrows indicate the right atrial ganglionated plexus, situated in the superior region adjacent to the roof of the right superior pulmonary vein (approximately 0.5-1 cm from the junction of the left atrium and the pulmonary vein roof): A) Target map for RAGP ablation (AP: RL); B) Target map for RAGP ablation (AP: PA)

**Ablation procedure:** due to the patient's intolerance to esophageal echocardiography, coronary computed tomography angiography (CTA) was performed to exclude left atrial thrombus. The findings were as follows: the left atrium appeared regular in morphology, with no evident thrombus. Following obtaining written informed consent, the patient was placed in a supine position, and both groin areas were routinely disinfected and draped. After local anesthesia with 1% lidocaine (100mg), a successful puncture of the left femoral vein was achieved, and a guidewire was inserted, followed by the placement of a 6F sheath with a hemostatic valve. With the sheath in place, electrodes were advanced along the femoral vein into the coronary sinus for controlled pacing. Subsequently, successful punctures were made at two sites in the right femoral vein, and a puncture needle was advanced along the femoral vein into the interatrial septum, with the successful passage of the guidewire and sheath into the left atrium. Pulmonary vein angiography was performed bilaterally, followed by the introduction of LASSO electrodes and ablation catheters, connected to the cardiac three-dimensional mapping system and the cooled saline irrigation ablation system, with concurrent administration of heparin for anticoagulation. Under the guidance of the three-dimensional mapping system, pulmonary vein isolation was performed on both left and right pulmonary veins, with a power setting of 30~35W and a temperature of 43°C, achieving successful isolation of pulmonary vein potentials where, AP signifies the anterior-posterior position, PA denotes the posterior-anterior position, RAO stands for right anterior oblique position, LAO represents left anterior oblique position, while RL and LL denote right and left side positions respectively (Figure 2). After pulmonary vein isolation, the patient's heart rhythm remained sinus bradycardia with a slow heart rate of 50 bpm. Subsequently, we performed ablation of the right atrial ganglionated plexus (RAGP). The right atrial ganglionated plexus (RAGP) is situated in the super anterior region adjacent to the ostium of the right superior pulmonary vein (approximately 0.5-1cm from the junction of the left atrium and the ostium of the pulmonary vein) and is easily exposed in the right anterior oblique projection (Figure 3); Here, RL denotes right side position, AP denotes anterior-posterior position, and PA signifies posterior-anterior position. The red arrow indicates the RAGP. During RAGP catheter ablation, an increase in heart rate can be observed within a few seconds, which is useful for confirming RAGP localization. Ablation therapy was performed using a contact force-sensing catheter (TactiCat™ Ablation Catheter, Sensor Enabled™, Abbott Medical (Shanghai) Co., Ltd.) in a power-controlled mode with a power limit set at 35W and a maximum temperature of 43°C. The catheter was continuously irrigated with saline at a rate of 17-25mL/min, and contact force was maintained between 10-20g throughout the ablation procedure. Atrial S1S1 pacing up to 240ms did not induce atrial fibrillation or atrial arrhythmias. The procedure was deemed successful, and the operation concluded with the timely removal of the sheath, local compression for hemostasis, and the patient experiencing no specific discomfort. The heart rate was 69 beats per minute, and blood pressure was 138/72mmHg. The patient was then safely returned to the ward for further observation, with sinus rhythm confirmed on electrocardiogram monitoring. The patient was advised to immobilize the right lower limb for 8 hours and received anticoagulation, and fluid replacement therapy, with attention given to immobilization of both lower limbs, wound bleeding, and peripheral circulation. Sinus rhythm was confirmed by electrocardiogram monitoring. The surgical procedure progressed smoothly without any patient-reported discomfort. Following three days of hospital observation, the patient was discharged.

### Discharge diagnosis and medications

**Discharge diagnosis:** 1) Paroxysmal atrial fibrillation; 2) Coronary atherosclerotic heart disease; 3) Insufficient cerebral artery blood supply; 4) Anxiety status; 5) Sinus bradycardia; 6) Coronary artery myocardial bridge; 7) Carotid artery plaque; 8) Pulmonary nodules.

**Medication instructions:** 1) Atorvastatin calcium tablets [20mg 7 tablets], oral, once nightly, 1 tablet per dose. 2) Famotidine tablets [10.5mg 20 tablets], oral, once daily, 0.5 tablet per dose. 3) Rivaroxaban tablets [15mg 7 tablets], oral, once daily, 15mg per dose. 4) Pantoprazole sodium enteric-coated capsules [40mg 7 granules], oral, once-daily before meals, 1 granule per dose. 5) Diclofenac potassium 400mg twice daily, 1 tablet per dose.

**Follow-up and outcomes:** in June 2023, the patient visited the outpatient clinic with no dizziness or syncope episodes. From June 6^th^ to 7^th^, 2023, dynamic electrocardiography revealed sinus node arrhythmia with irregularities, short paroxysmal atrial tachycardia, atrial premature beats (710 times), and ST-segment changes. Over 24 hours, the total number of heartbeats was 69,631, with a minimum of 41bpm (June 7, 2023, 03: 34: 47), a maximum of 52 bpm (June 7, 2023, 12: 04: 34), and an average of 50bpm, with no prolonged pauses. In January 2024, the patient visited the outpatient clinic with no episodes of dizziness or syncope. On January 15, 2024, the electrocardiogram showed sinus rhythm with left axis deviation and a heart rate of 63bpm. From January 16^th^ to 17^th^, dynamic electrocardiography revealed sinus bradycardia with irregularities and atrial premature beats (219 times) over 24 hours, with a total of 69,990 heartbeats, ranging from a minimum of 40bpm (January 19, 2023, 02: 03: 29) to a maximum of 71bpm (January 18, 2023, 14: 57: 33), averaging 55bpm, with the longest RR interval of 1.85 seconds. The echocardiogram indicated a left atrial diameter of 36mm, left ventricular diastolic diameter of 51mm, left ventricular systolic diameter of 30mm, interventricular septum thickness of 9mm, left ventricular posterior wall thickness of 9mm, and left ventricular ejection fraction of 71%.

**Patient perspective:** the patient was delighted with the quality of care.

**Informed consent:** written informed consent was obtained from the patient for the publication of this case report.

## Discussion

Sinus bradycardia is a common clinical finding in patients with Atrial Fibrillation (AF), often presenting asymptomatically, and is particularly prevalent among the elderly population. The administration of Antiarrhythmic Drugs (AADs) in these patients necessitates caution due to the risk of further heart rate reduction and the potential impact on hemodynamic stability. This clinical challenge requires electrophysiologists to carefully weigh the benefits and risks of AADs against each patient's unique condition and clinical requirements, guiding treatment decisions based on experience and clinical guidelines. This case report examines combined AF radiofrequency and cardiac neural ablation effects on a patient with paroxysmal AF and sinus bradycardia. Following circumferential pulmonary vein isolation, ablation of the RAGP was conducted, preceding AAD administration. Given amiodarone's lengthy half-life and propafenone's inapplicability, we selected dronedarone with a shorter half-life, administered at 400mg bid po.

The cardiac autonomic nervous system, consisting of sympathetic and parasympathetic nerves, regulates heart rate and contractility through distinct neurotransmitters. The sympathetic nervous system elevates heart rate and contractility by releasing adrenaline and noradrenaline, while the parasympathetic nervous system counters with acetylcholine to decelerate heart rate and diminish contractility [4]. Cardiac neural ablation adjusts this autonomic balance, offering a potential treatment for certain arrhythmias. However, this intervention's specific mechanisms and long-term efficacy await further research. Cardiac neural ablation primarily targets the intrinsic autonomic ganglia, particularly the vagal ganglia, to address arrhythmias such as AF and vasovagal syncope [5]. Under normal conditions, these systems maintain heart rate and function through interactive balance. During ablation, specific cardiac ganglia are targeted to mitigate excessive vagal influence, essential for treating vagally mediated arrhythmias [6]. For example, ablation can prevent vasovagal syncope, where excessive vagal activity precipitates a sudden drop in heart rate and blood pressure, leading to fainting. While this reduces vagal activity and increases heart rate, the potential for sympathetic dominance and heart rate acceleration exists. It is crucial to acknowledge that the long-term efficacy and safety of cardiac neural ablation are yet to be confirmed through extensive clinical trials [7]. Variability in localization and ablation strategies for autonomic ganglia across institutions may influence surgical outcomes [8]. Moreover, post-ablation nerve reconnection could impact long-term results.

At least seven intrinsic autonomic ganglia within the heart are targeted in cardiac neural ablation, with five key locations surrounding the pulmonary veins in the left atrium. These include the left superior, inferior, and lateral ganglia, as well as the right anterior and inferior ganglia [9]. Standardization is lacking in the localization and ablation of these ganglia, leading to varied approaches among medical institutions that may affect treatment consistency [10]. Notably, the RAGP is a critical target for treating vasovagal syncope and bradyarrhythmias. This ablation site, located anterior to the right pulmonary vein orifice, can significantly elevate heart rate during surgery and offers effective short- and long-term therapeutic benefits, reducing syncope recurrence. In this study, the patient experienced sinus bradycardia before and after AF radiofrequency ablation. After undergoing cardiac neural ablation, specifically right anterior ganglion ablation, the patient was treated with AADs, with safety monitored throughout. Post-AAD administration, we assessed indicators such as bradycardia severity, symptom improvement, and drug tolerance. A 10-month follow-up revealed good AAD tolerance, with no significant adverse events.

## Conclusion

In summary, this study's findings indicate the potential safety of AAD use in patients with sinus bradycardia following AF radiofrequency ablation and cardiac neural ablation. These results offer valuable insights for clinical decision-making. However, given the single-patient sample, further research is warranted to substantiate these findings. The case provides preliminary evidence supporting the safety of AADs in this patient population, aiding clinicians in treatment selection. The outcomes underscore the approach's feasibility and efficacy, suggesting its potential application in clinical practice.
